# Full-Field Electroretinographic Evaluation of a Case With Benign Lobular Inner Nuclear Layer Proliferations of the Retina

**DOI:** 10.7759/cureus.95500

**Published:** 2025-10-27

**Authors:** Mizuki Yamauchi, Ken Fukuda, Tomoka Mizobuchi, Isana Nakajima, Kenji Yamashiro

**Affiliations:** 1 Ophthalmology, Kochi Medical School, Kochi, JPN

**Keywords:** benign lobular inner nuclear layer proliferations, congenital hypertrophy of the retinal pigment epithelium, electroretinography, optical coherence tomography, retinal tumor

## Abstract

Benign lobular inner nuclear layer proliferation (BLIP) is a newly recognized retinal tumor defined by a benign, non‐progressive lesion confined to the inner nuclear layer. Although several cases have been reported worldwide, its functional impact on the retina remains unclear, as prior studies have focused mainly on structural imaging. We report a seven-year-old Japanese boy with BLIP associated with congenital hypertrophy of the retinal pigment epithelium (CHRPE). Multimodal imaging revealed typical intraretinal lesions, and full-field electroretinography (ERG) demonstrated preserved scotopic and photopic responses. To our knowledge, this is the first report of full-field ERG performed in a patient with BLIP, showing that overall retinal function can remain intact.

## Introduction

Benign lobular inner nuclear layer proliferation (BLIP) is a newly described clinical entity defined by a benign retinal tumor arising within the retina’s inner nuclear layer. BLIP was first reported in 2023 by Sanfilippo et al. in four patients aged 5-32 years [1]. BLIP is considered a benign intraretinal tumor that does not show growth or invasive features and is often found incidentally on fundus examination in children or young adults, suggesting that it may be congenital. To date, 22 cases of BLIP have been reported in the literature [1-10]. In addition, previous reports have noted a frequent association between BLIP and congenital hypertrophy of the retinal pigment epithelium (CHRPE), although the exact relationship remains unclear. Importantly, while prior studies have focused on structural characteristics, the functional impact of BLIP on the retina has rarely been evaluated with electroretinography (ERG) or other functional testing. Herein, we report a case of a seven-year-old Japanese boy with BLIP associated with CHRPE, representing the first case in which retinal function was assessed by full-field ERG.

## Case presentation

**Figure 1:**
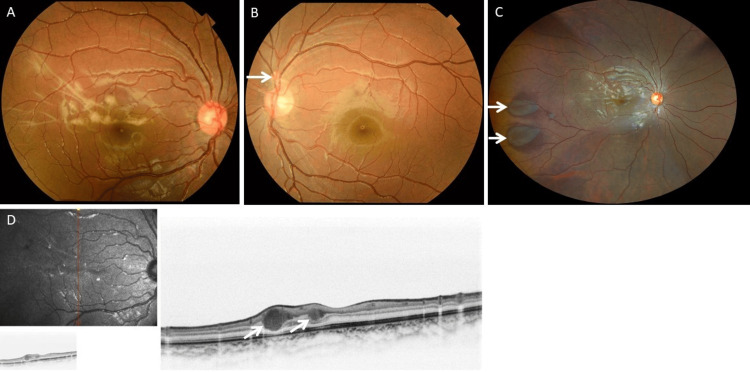
Fundus photographs and optical coherence tomography images Fundus photographs show a multifocal white intraretinal mass at the posterior pole in the right eye (A) and a lesion above the optic nerve in the left eye (B). A wide-field fundus photograph shows multifocal CHRPE in the temporal peripheral retina of the right eye (C). Optical coherence tomography of the right eye shows hyperreflective lesions within the inner nuclear layer, and the lesions were partially indistinct at the interface of the inner plexiform layer (D). CHRPE, congenital hypertrophy of the retinal pigment epithelium

**Figure 2:**
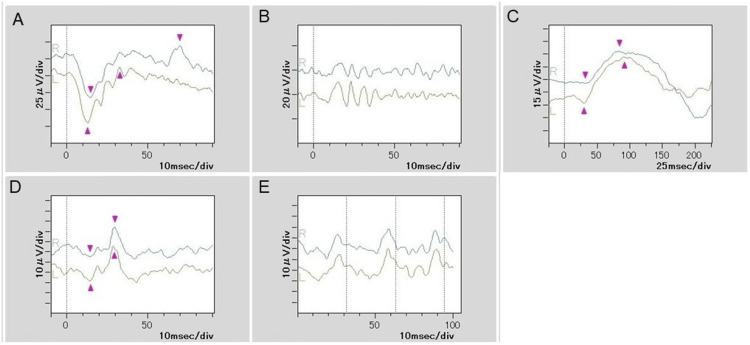
Electroretinogram ERG shows normal amplitudes in both eyes. (A) Dark-adapted 3.0 ERG (combined rod–cone response). (B) Dark-adapted 3.0 oscillatory potentials. (C) Dark-adapted 0.01 ERG (rod response). (D) Light-adapted 3.0 ERG (cone response). (E) Light-adapted 30 Hz flicker ERG. ERG, electroretinography

An asymptomatic seven-year-old boy presented to an ophthalmologist for an eye examination. He was born full-term and had no significant medical or ocular history. His best corrected visual acuity was 20/16 (-0.1 logMAR) OD and 20/12 (-0.2 logMAR) OS. Slit-lamp examination revealed no remarkable changes in the ocular surface bilaterally. Inflammatory cells in the anterior chamber and lens opacity were not observed in either eye. Fundus examination demonstrated a multifocal white intraretinal mass with arching extensions at the posterior pole in the right eye (Figure 1A). A similar lesion was also observed above the optic disc in the left eye (Figure 1B). In the peripheral retina of the right eye, multifocal pigmented lesions consistent with CHRPE were also observed (Figure 1C). Optical coherence tomography revealed hyper-reflective lesions in the inner nuclear layer (Figure 1D). The lesions were partially indistinct at the interface of the inner plexiform layer, which suggests the possibility of partial infiltration into that layer. Full-field ERG (HE-2000, TOMEY, Japan) was performed in accordance with the International Society for Clinical Electrophysiology of Vision (ISCEV) standard protocol. Although ERG showed no obvious abnormality in scotopic or photopic function in either eye (Figure 2), right oscillatory potentias (OPs) appear slightly reduced compared to left (Figure 2B). These retinal lesions remained stable without visual impairment over a year of follow-up.

## Discussion

To date, only a few intrinsic neoplasms have been reported to originate from the neurosensory retina, including astrocytic hamartoma, retinal astrocytoma, and retinoblastoma. More recently, another entity - BLIP - has been proposed. To date, 22 cases of BLIP have been reported in the literature [1-10]. These reports have originated from the United States, United Kingdom, Belgium, France, Colombia, Turkey, and Japan. As far as we are aware, this represents the first documented case of BLIP including a full-field electroretinographic assessment.

Although previous reports focused mainly on structural evaluation, no reports included retinal functional tests such as ERG. This is the first report of full-field ERG assessment in a patient with BLIP, demonstrating normal retinal function. Our findings suggest that BLIP does not affect overall retinal function. However, full-field ERG provides a summed electrical response from the entire retina and may fail to detect subtle, localized dysfunction caused by a small intraretinal lesion. A multifocal ERG (mfERG) or focal ERG, which assesses localized retinal areas, would be more sensitive for detecting focal retinal deficits in such cases. In the report by Kogo et al., focal macular ERG testing demonstrated that although the a- and b-wave amplitudes were preserved, the OPs were reduced, and the OP-to-a-wave amplitude ratio was decreased. This pattern suggests a selective functional impairment of inner retinal interneurons, particularly amacrine cells. In our patient, the full-field ERG showed no apparent abnormalities; however, the OPs in the right eye were mildly reduced compared with those in the left eye. This finding is consistent with the pattern described by Kogo et al. [10]. In this case, visual field testing could not be performed due to the patient’s young age; nevertheless, it should be included in the evaluation of retinal function in this disease.

BLIP is, therefore, usually found incidentally in asymptomatic young, healthy patients. Our patient was a seven-year-old boy with no symptoms and good visual acuity. The lesion was discovered during a routine eye examination, which is consistent with previously reported cases. BLIP is likely a non-progressive lesion; a 55-year-old patient with BLIP reportedly showed no change in the lesion over 30 years [2]. In our case, the lesion remained unchanged during approximately one year of follow-up. The patient remains asymptomatic with preserved vision. However, further studies are needed to determine whether visual function and retinal integrity remain unchanged over the long term in patients with BLIP.

As described in the report by Sanfilippo et al., BLIP can be associated with CHRPE [1]. In previously reported cases, CHRPE was observed in 16 of 22 patients with BLIP. Our case also showed multifocal CHRPE in the right eye but not in the left eye. Although frequent association of BLIP and CHRPE may suggest they share a similar developmental origin, the association between BLIP and CHRPE is still unclear.

## Conclusions

We report the first case with BLIP wherein retinal function was evaluated using full-field ERG. Our findings demonstrate preserved retinal function and support the concept that BLIP is a stable and asymptomatic condition. However, multimodal imaging and functional examinations such as ERG should be performed for further understanding of the pathogenesis and clinical characteristic patterns of BLIP during diagnosis, management, and follow-up of such tumors.

## References

[1] Sanfilippo CJ, Javaheri M, Handler S (2023). Benign lobular inner nuclear layer proliferations of the retina associated with congenital hypertrophy of the retinal pigment epithelium. Ophthalmology.

[2] Shah M, Charbel Issa P (2024). Long-term stability of benign lobular inner nuclear layer proliferations. JAMA Ophthalmol.

[3] Boca T, Cohen SY, Hermouet E, Srour M, Miere A (2025). Benign lobular inner nuclear layer proliferation associated with congenital hypertrophy of the retinal pigment epithelium. Retin Cases Brief Rep.

[4] Pastor-Idoate S, Heimann H, Keane PA, Balaskas K, Lujan BJ (2016). Diagnostic and therapeutic challenges. Retina.

[5] Sekeroglu MA, Tekin K (2025). Benign lobular inner nuclear layer proliferations of the retina. Ophthalmology.

[6] Javaheri M, Sanfilippo CJ, Kasi SK (2025). Phenotypic spectrum of benign lobular inner nuclear layer proliferations: a multicenter analysis and review of the literature. Ophthalmol Retina.

[7] Córdoba-Ortega CM, Arias Aristizabal JD, Gómez Velasco MA, Martinez Pulgarín DF (2025). Benign lobular inner nuclear layer proliferations of the retina. Eur J Ophthalmol.

[8] Yalcinbayir O, Ucan Gunduz G, Yalcinbayir D (2025). Benign lobular inner nuclear layer proliferations. Ophthalmol Retina.

[9] Ambresin A, Barbosa M (2023). Ultra-widefield image of the month: benign lobular inner nuclear proliferations (BLIPs) of the retina associated with congenital hypertrophy of the retinal pigment epithelium. Retin Physician.

[10] Kogo T, Muraoka Y, Nishigori N (2025). Multimodal analysis of benign lobular inner nuclear layer proliferation. Ophthalmol Retina.

